# Laparoscopic versus open surgery for gallbladder carcinoma: safety, feasibility, and oncological outcomes

**DOI:** 10.1007/s12094-023-03207-4

**Published:** 2023-04-28

**Authors:** Xin Wu, Binglu Li, Chaoji Zheng, Wei Liu, Tao Hong, Xiaodong He

**Affiliations:** Department of General Surgery, Peking Union Medical College Hospital, Chinese Academy of Medical Sciences and Peking Union Medical College, No. 1 Shuaifuyuan, Dongcheng District, Beijing, 100730 China

**Keywords:** Gallbladder carcinoma, Laparoscopic surgery, Open surgery, Propensity score matched analysis, Kaplan–Meier curves

## Abstract

**Background:**

Gallbladder carcinoma (GC) is a rare malignant tumor. Laparoscopic technology has revolutionized the reality of surgery. However, whether laparoscopic surgery is suitable for GC has not been clarified. We aimed to analyze the safety, feasibility, and oncological outcomes of laparoscopic surgery in GC.

**Methods:**

The medical records of patients with GC treated at our hospital between January 2016 and December 2021 were retrospectively reviewed. Patients who underwent laparoscopic and open surgery were compared. Propensity score matched analysis was performed to balance the basic characteristics of the two groups. Kaplan–Meier curves were used to describe and compare the overall and disease-free survival rates between the groups.

**Results:**

A total of 163 patients with GC were included. Cholelithiasis was detected in 64 (39.3%) patients. Seventy patients were matched after propensity score matching. The laparoscopic group was significantly better than the open group in terms of operation time (p < 0.001), blood loss (p = 0.002), drain time (p = 0.001), and hospital stay (p < 0.001). After a median follow-up time of 19 (12, 35) months, there was no significant difference in the cumulative overall (p = 0.650) and disease-free (p = 0.663) survival rates between the laparoscopic and open groups according to Kaplan–Meier curves.

**Conclusion:**

Laparoscopic surgery can reduce the operation time and blood loss, and shorten drain time and hospital stay without increasing the incidence of complications. Patients undergoing laparoscopic and open surgery have a similar prognosis. Laparoscopic surgery is worth promoting in patients with GC.

## Introduction

Biliary tract carcinomas include tumors of the bile duct and gallbladder [1]. Compared to the bile duct cancer, gallbladder carcinoma (GC) is often asymptomatic in the early stage, making diagnosis and treatment more difficult. GC is relatively rare, accounting for only 0.6–1.2% of all tumors and 0.9–1.7% of all tumor-related deaths [2, 3]. Its incidence varies greatly among different countries and regions, with Asia having the highest prevalence [4]. It is estimated that there will be approximately 12,130 new cases and 4400 deaths from gallbladder and extrahepatic bile duct carcinomas in the United States in 2022 [5]. However, the estimated number of new cases and deaths only from GC reached 52,800 and 40,700, respectively, in China as early as 2015 [6].

Surgery plays a key role in the comprehensive treatment of GC. It is the only potential way to cure this rare malignant disease [4, 7, 8]. However, more than 90% of symptomatic and 80% of asymptomatic GC patients are already at an advanced stage at the time of diagnosis [4]. Surgery can be therapeutic in some patients, but is only a tumor staging modality in others [8]. Laparoscopic technology has revolutionized the reality of surgery since its inception. It has had a huge impact on general surgery, thoracic surgery, urology, and gynecology. Laparoscopic surgery (LS) has also developed well in the field of bile duct surgery since it was widely used to remove the gallbladder in the 1990s. However, the debate on whether LS can lead to tumor spread and inadequate curative resection has never ceased [9]. The Japanese Society of Hepato-Biliary-Pancreatic Surgery even strongly recommends open surgery for suspected GC [10]. At the same time, many studies have shown that LS is feasible in GC [11, 12]. The present study aimed to analyze the safety, feasibility, and oncological outcomes of LS in GC, so as to provide recommendations for the treatment of this malignant disease.

## Methods

### Patients

This is a retrospective cohort study based on propensity score matched analysis. Two independent surgeons retrospectively reviewed the medical records of patients with GC admitted to Peking Union Medical College Hospital between January 2016 and December 2021. Patients were included in the present study if they met the following inclusion criteria: (1) GC was diagnosed by paraffin pathology postoperatively; (2) patients underwent radical surgery in our hospital; (3) medical records were complete. Patients were excluded if they satisfied the following exclusion criteria: (1) pathological examination revealed neuroendocrine neoplasm, squamous cell carcinoma, and other rare neoplasms; (2) patients had other digestive system tumors. A database containing the general characteristics, clinical manifestations, examination results, surgical details, pathological results, and follow-up information was established. Disputed data were resolved by discussion. The study was reviewed and approved by the Peking Union Medical College Hospital Institutional Review Board.

### Treatment and follow up

All patients underwent liver function tests, ultrasonography, and computed tomography (CT) before surgery. Positron emission computed tomography was not used routinely due to its price, and only 23 patients underwent this test before surgery in the present study. GC diagnosed after cholecystectomy was defined as incidental GC. Postoperative complications were defined as any deviation from the normal recovery course that occurred within 30 days after surgery and classified by the Clavien–Dindo system [13]. Adjuvant therapy was recommended for patients with positive lymph node or stage T2 and above. Patients were followed up every three months for the first year after discharge, and every six months thereafter. Blood tests, ultrasonography, and CT were used for follow-up. CT results were used as the basis for judging tumor recurrence and metastasis, and pathological results were not required.

### Surgical principle and laparoscopic technique

The operation was performed under general anesthesia and in the supine and reverse Trendelenburg positions for open surgery and LS, respectively. For patients diagnosed with GC preoperatively and intraoperatively, after performing intraoperative frozen diagnosis and preliminary staging, the scope of the surgery was determined according to the different T staging. Patients with T1 stage disease were recommended to undergo cholecystectomy and regional lymph node dissection. Patients with T2 stage and above were recommended to undergo cholecystectomy, regional lymph node dissection, and partial liver resection. For patients with right hepatic artery or cystic duct involvement, right hemihepatectomy or bile duct resection was performed, respectively. For patients diagnosed with GC incidentally after cholecystectomy, only those with T1a stage and without cystic lymph node metastasis received close follow-up, while others were recommended to undergo radical reoperation. The scope of regional lymph node dissection included groups 8, 12, and 13 lymph nodes. Partial liver resection comprised liver wedge resection (at least 2 cm margin from the gallbladder bed) and segment 4b + 5 resection. The actual surgical extent was determined according to the tumor stage, patients' general health condition and willingness. Drainage tubes were routinely placed in patients underwent radical surgery. The surgeons included in the study had similar experience in all cases.

**Fig. 1:**
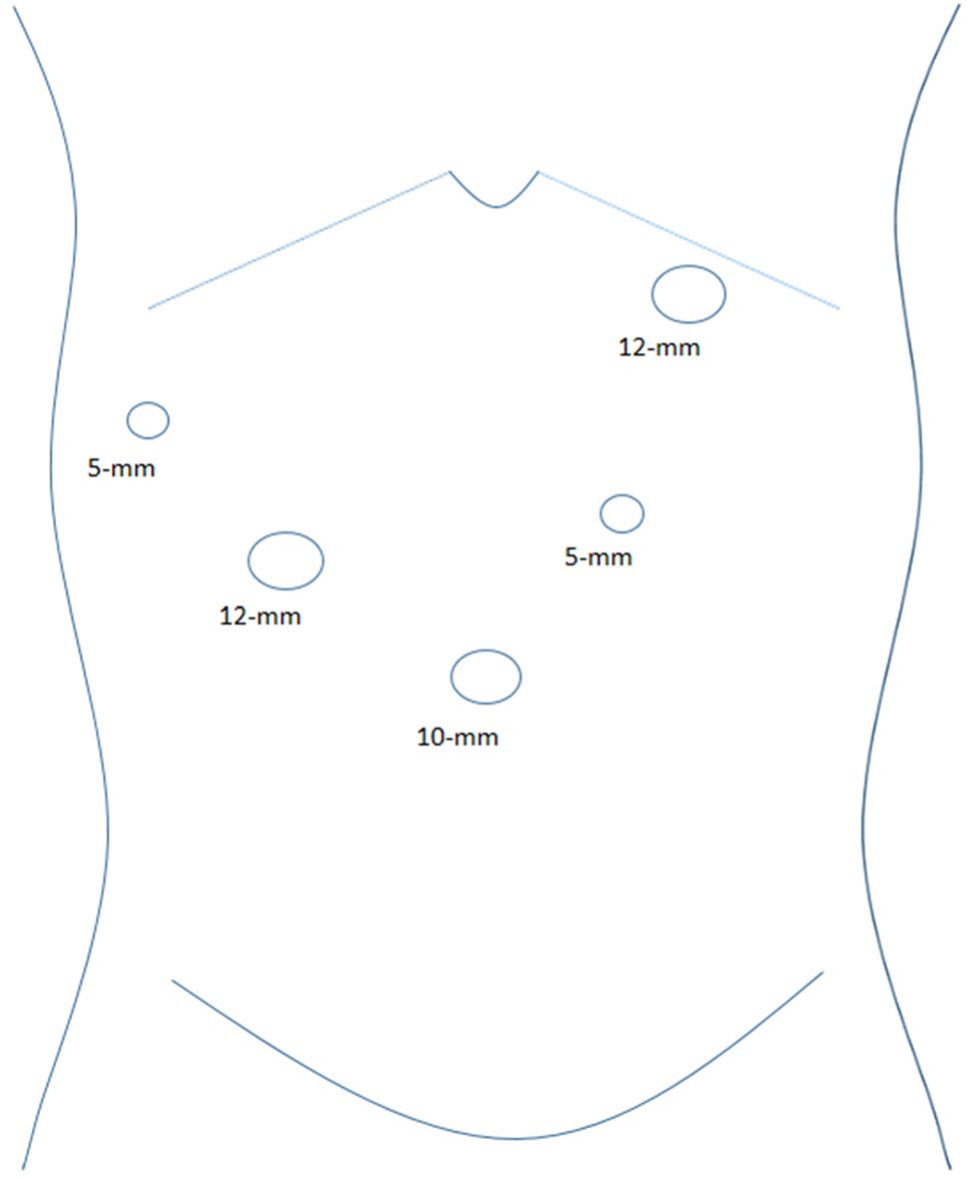
Placement of trocars in the laparoscopic surgery for gallbladder carcinoma. The operator was positioned on the patient's right and the assistant was on the left. Each person had a 12-mm trocar and a 5-mm trocar for operation. The 10-mm trocar was for the scopiest who was positioned between the patient’s legs

Laparoscopic and open surgery were recommended to different patients based on their preoperative imaging results and willingness. LS was only performed in patients who were willing to undergo this pattern and did not have extrahepatic organ involvement. The operator, assistant, and scopiest were positioned on the patient's right, left, and between the patient’s legs, respectively. Pneumoperitoneum was established through a 10-mm trocar which was placed at the umbilicus. Four additional trocars (two 12-mm trocars and two 5-mm trocars) were then placed. The location of trocars are shown in Fig. 1. The intra-abdominal pressure was maintained around 12–15 mmHg. Cholecystectomy was performed firstly and a retrieval bag was used to collect specimen. After GC was diagnosed by intraoperative frozen, lymph node dissection and partial liver resection would be performed.

### Statistical analysis

All analyses were carried out through the Statistical Package for Social Sciences software 25.0 (IBM Corp., Armonk, NY, USA). Categorical variables were shown as absolute numbers and frequencies. Continuous variables with normal and skewed distribution were presented as mean ± standard deviation and median (25th, 75th), respectively. Differences between groups were analyzed using the t-test, Mann–Whitney U test, χ^2^ test, or Fisher’s exact test, as appropriate. Propensity score matched analysis was performed to match LS and open surgery in a 1:1 manner using the nearest neighbor matching. The match tolerance was 0.02. The cumulative survival rates were described and analyzed by Kaplan–Meier curves with the log-rank test. Statistical significance was set at p < 0.05.

## Results

According to the inclusion and exclusion criteria, a total of 163 patients with GC who were treated in Peking Union Medical College Hospital from January 2016 to December 2021 were included. There were 73 (44.8%) men and 90 (55.2%) women, with a mean age of 63.8 ± 9.7 years. Abdominal discomfort was the most common preoperative symptom, occurring in 80 (49.1%) patients. Cholelithiasis was detected in 64 (39.3%) patients. All patients underwent surgery and the mean operation time was 177.1 ± 76.5 min, of which 125 (76.7%) patients underwent liver resection. Four patients underwent right hemihepatectomy and 18 patients underwent bile duct resection. A total of 18 patients received blood transfusion. They all underwent open surgery, and the median blood transfusion volume was 4 (2, 6) U of erythrocyte and 400 (400, 800) mL of plasma. Patients were divided into two groups according to the operation method, the laparoscopic group (n = 44) and open group (n = 119). The general information, symptoms, underlying diseases, and laboratory results of the two groups are compared in Table 1. LS and open surgery did not show significant differences in these respects. The surgical details and pathological information are compared in Table 2. The laparoscopic group was superior to the open group in the operation time (p < 0.001), blood loss (p < 0.001), complications (p = 0.039), drain time (p < 0.001), and hospital stay (p < 0.001). However, fewer patients in the laparoscopic group underwent liver resection (p = 0.005), and the T (p = 0.027), N (p = 0.003), and TNM (p = 0.017) stages were less severe.

**Table 1:** Comparison of general data of patients who underwent laparoscopic and open surgery

	Laparoscopic surgery (n = 44)	Open surgery (n = 119)	p
Sex (male/female) (n)	19/25	54/65	0.802
Age (year)	66.0 ± 10.3	63.0 ± 9.3	0.076
Body mass index (kg/m^2^)	24.4 ± 3.5	23.8 ± 3.4	0.347
Abdominal discomfort (n)	24	56	0.396
Jaundice (n)	2	10	0.617
Fever (n)	4	5	0.408
Cholelithiasis (n)	20	44	0.325
Hypertension (n)	14	49	0.276
Diabetes (n)	8	18	0.636
Smoking (n)	8	36	0.123
Drinking (n)	7	23	0.617
CEA > 5 ng/mL	8	16	0.449
CA19-9 > 34 U/mL	9	42	0.070
ALT > 40 U/L	3	16	0.242
Albumin < 35 g/L	2	2	0.295
Total bilirubin > 22.2 µmol/L	5	17	0.628
Direct bilirubin > 6.8 µmol/L	6	22	0.466

*CEA* carcinoembryonic antigen, *CA19-9* carbohydrate antigen 19–9, *ALT* alanine aminotransferase

**Table 2:** Comparison of the surgical details and pathological information of patients who underwent laparoscopic and open surgery

	Laparoscopic surgery (n = 44)	Open surgery (n = 119)	p
Operation time (min)	120.3 ± 69.5	198.2 ± 68.1	< 0.001
Blood loss (mL)	50 (50, 100)	200 (100, 300)	< 0.001
ASA ≥ III (n)	7	19	0.993
Surgical extent (n)			< 0.001
Cholecystectomy	12	1	
Cholecystectomy + LND	5	20	
Cholecystectomy + LND + LWR	12	38	
Cholecystectomy + LND + LSR	15	60*	
Liver resection (n)	27	98	0.005
Bile duct resection (n)	4	14	0.840
Incidental gallbladder carcinoma	3	26	0.026
Complications (n)	6	35	0.039
Clavien–Dindo I/II/IIIa/V	1/4/1/0	12/18/4/1	0.740
Drain time (n)	4 (3, 5)	6 (5, 8)	< 0.001
Hospital stay (n)	8 (7, 11)	14 (11, 16)	< 0.001
Tumor size ≥ 2 cm (n)	14	43	0.608
Positive margin (n)	2	6	1
T stage (1/2/3/4) (n)	11/21/12/0	13/47/53/6	0.027
N stage (0/1/2/X) (n)	39/2/2/1	76/33/8/2	0.003
TNM stage (n)			0.017
I	11	13	
II	18	35	
III	10	55	
IV	5	16	

*ASA* American Society of Anesthesiologists, *LND* lymph node dissection, *LWR* liver wedge resection, *LSR* liver segment 4b + 5 resection

*Including 4 patients underwent right hemihepatectomy

**Fig. 2:**
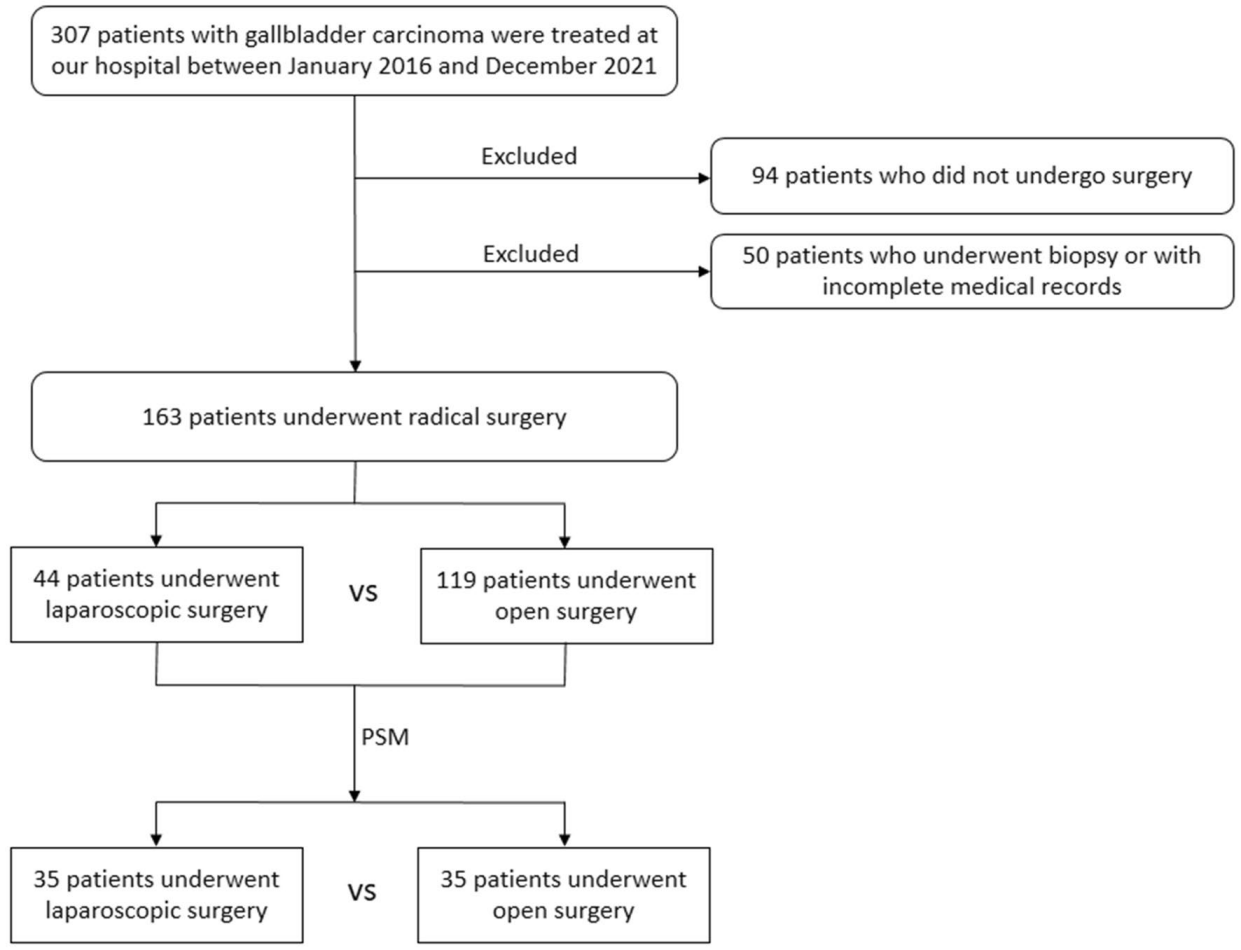
The flow diagram of the study participant selection. 163 and 70 patients with gallbladder carcinoma were included before and after propensity score matched analysis, respectively

To better compare the two groups, propensity score matched analysis was performed. Patients who underwent LS were matched 1:1 with those who underwent open surgery. The sex, age, body mass index, cholelithiasis, liver resection, and TNM stage were used as predictors for the matching. In total, 35 pairs of patients were matched. The flow diagram of the study participant selection is shown in Fig. 2. The two groups showed good consistency in general information, symptoms, underlying diseases, laboratory results, liver resection, complications, and pathological details (Tables 3, 4). The laparoscopic group was still significantly better than the open group in terms of operation time (p < 0.001), blood loss (p = 0.002), drain time (p = 0.001), and hospital stay (p < 0.001) (Table 4).

**Table 3:** Comparison of the general data of patients who underwent laparoscopic and open surgery after propensity score matching

	Laparoscopic surgery (n = 35)	Open surgery (n = 35)	p
Sex (male/female) (n)	14/21	13/22	0.806
Age (year)	66.1 ± 9.6	64.3 ± 11.8	0.494
Body mass index (kg/m^2^)	24.3 ± 3.6	23.4 ± 3.3	0.283
Abdominal discomfort (n)	21	15	0.151
Jaundice (n)	1	2	1
Fever (n)	2	1	1
Cholelithiasis (n)	16	14	0.629
Hypertension (n)	13	14	0.806
Diabetes (n)	7	5	0.526
Smoking (n)	7	10	0.403
Drinking (n)	5	7	0.526
CEA > 5 ng/mL	7	8	0.771
CA19-9 > 34 U/mL	9	14	0.203
ALT > 40 U/L	3	3	1
Albumin < 35 g/L	2	1	1
Total bilirubin > 22.2 µmol/L	3	5	0.710
Direct bilirubin > 6.8 µmol/L	5	7	0.526

*CEA* carcinoembryonic antigen, *CA19-9* carbohydrate antigen 19–9, *ALT* alanine aminotransferase

**Table 4:** Comparison of the surgical details and pathological information of patients who underwent laparoscopic and open surgery after propensity score matching

	Laparoscopic surgery (n = 35)	Open surgery (n = 35)	p
Operation time (min)	128.3 ± 62.9	195.1 ± 57.8	< 0.001
Blood loss (mL)	50 (50, 100)	100 (50, 200)	0.002
ASA ≥ III (n)	6	6	1
Surgical extent (n)			0.464
Cholecystectomy	4	1	
Cholecystectomy + LND	4	7	
Cholecystectomy + LND + LWR	12	10	
Cholecystectomy + LND + LSR	15	17	
Liver resection (n)	27	27	1
Bile duct resection (n)	3	3	1
Incidental gallbladder carcinoma	3	2	1
Complications (n)	5	8	0.356
Clavien–Dindo I/II/IIIa	1/3/1	2/4/2	1
Drain time (n)	4 (3, 5)	5 (4, 8)	0.001
Hospital stay (n)	8 (7, 11)	12 (10, 15)	< 0.001
Tumor size ≥ 2 cm (n)	14	13	0.806
Positive margin (n)	2	1	1
T stage (1/2/3/4) (n)	4/19/12/0	3/17/13/2	0.645
N stage (0/1/2/X) (n)	30/2/2/1	26/6/2/1	0.565
TNM stage (n)			0.751
I	4	3	
II	16	15	
III	10	14	
IV	5	3	

*ASA* American Society of Anesthesiologists, *LND* lymph node dissection, *LWR* liver wedge resection, *LSR* liver segment 4b + 5 resection

**Fig. 3:**
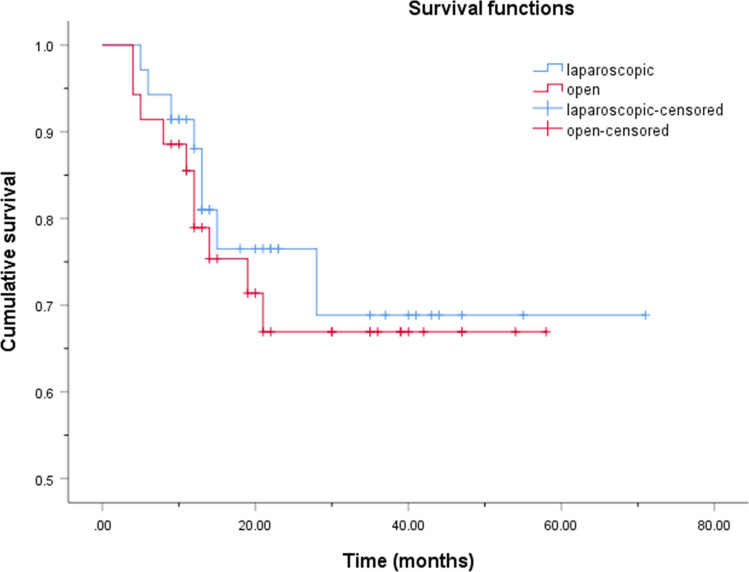
Kaplan–Meier cumulative overall survival curves for gallbladder carcinoma patients who underwent laparoscopic and open surgery. Patients who underwent laparoscopic and open surgery showed no significant difference in the cumulative overall survival rates (log-rank, p = 0.650)

**Fig. 4:**
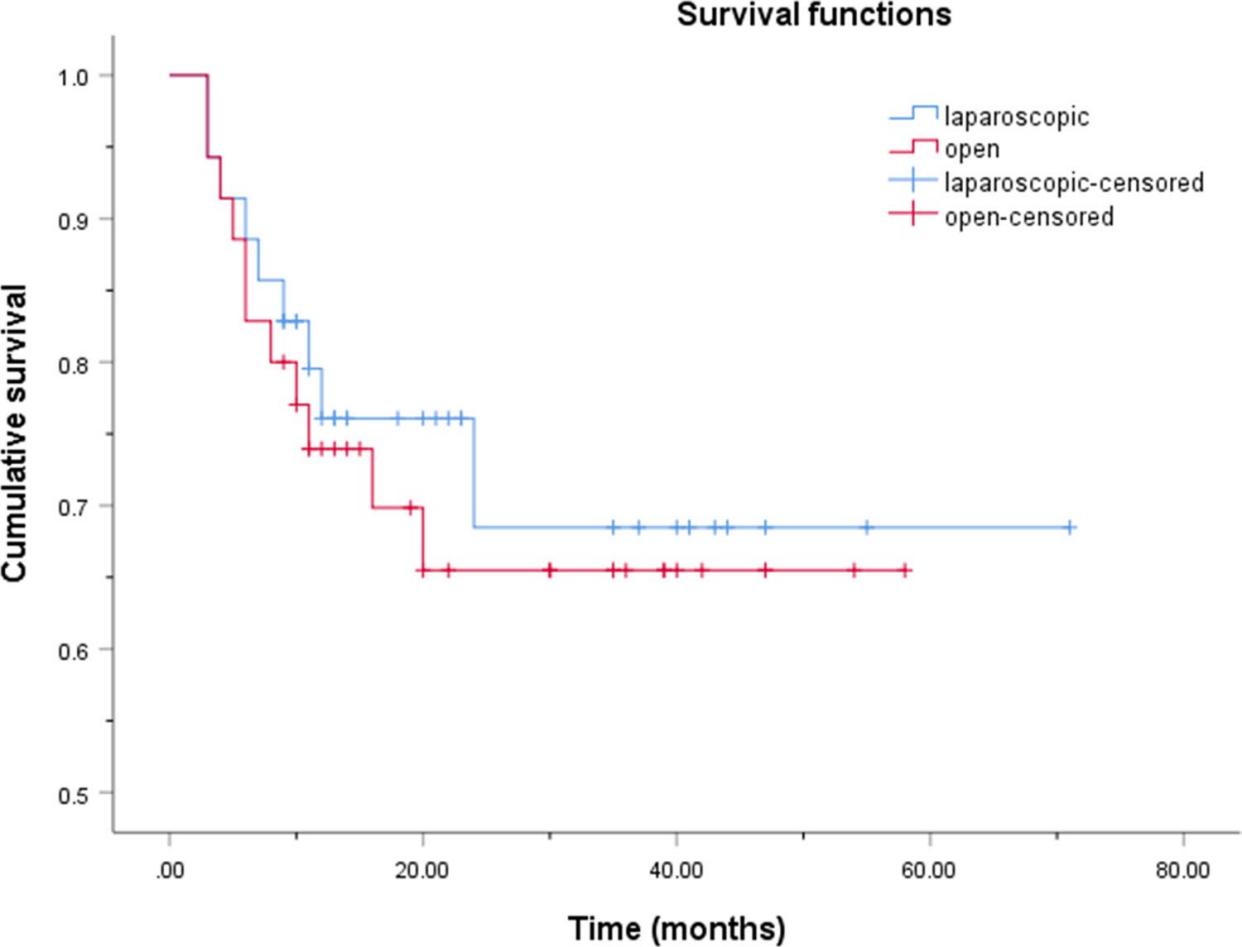
Kaplan–Meier cumulative disease-free survival curves for gallbladder carcinoma patients who underwent laparoscopic and open surgery. Patients who underwent laparoscopic and open surgery showed no significant difference in the cumulative disease-free survival rates (log-rank, p = 0. 663)

As of July 2022, all 70 matched patients were followed up for a median time of 19 (12, 35) months. Among them, 50 patients survived without a tumor, two patients survived with a tumor, and 18 patients died. Two patients survived with a tumor all had retroperitoneal lymph node metastases. Of the 18 patients who died, 10 had hepatic metastases, 7 had hepatic and retroperitoneal lymph node metastases, and 1 had hepatic and cardiophrenic angle lymph node metastases. Adjuvant chemotherapy and targeted therapy were recommended for patients with recurrence. Radiofrequency ablation was also recommended for patients with hepatic metastases. Kaplan–Meier curves were used to describe the cumulative survival rates. The 1- and 3- year cumulative overall survival (OS) rates of laparoscopic group were 88.0% and 68.8%, respectively. For the open group, they were 78.9% and 66.9%. The 1- and 3- year cumulative disease-free survival (DFS) rates of laparoscopic group were 76.1% and 68.5%, respectively. For the open group, they were 74.0% and 65.5%. There was no significant difference in the cumulative OS (Fig. 3, p = 0.650) and DFS (Fig. 4, p = 0.663) rates between patients who underwent LS and open surgery. Several previous studies with propensity score matched analysis are compared with the present study in Table 5. All studies suggested that there was no significant difference in prognosis between LS and open surgery.

**Table 5:** Comparison between the present study and several previous studies with propensity score matched analysis

No.	1 (our study)	2 [9]	3 [15]	4 [22]
First author (year)	Wu (2023)	Cho (2022)	Kim (2021)	Navarro (2020)
Country	China	Korea	Korea	Korea
Number of patients (n)				
Laparoscopic	35	19	17	43
Open	35	19	17	43
Operation time (min)				
Laparoscopic	128.3 ± 62.9	218.9 ± 145.0	175(160, 180)	139.1 ± 97.1
Open	195.1 ± 57.8	316.8 ± 80.3	156(120, 191)	211.2 ± 91.4
p	< 0.001	0.016	0.370	0.001
Blood loss (mL)				
Laparoscopic	50 (50, 100)	–	300(300, 500)	71.6 ± 178.8
Open	100 (50, 200)	–	300(200, 900)	208.1 ± 242.2
p	0.002	–	0.846	0.004
Hospital stays (day)				
Laparoscopic	8 (7, 11)	8.4 ± 5.9	7.0(7.0, 9.0)*	6.1 ± 9.8*
Open	12 (10, 15)	14.4 ± 6.0	12.0(10.0, 14.0)*	12.6 ± 5.5*
p	< 0.001	0.004	0.009	0.0001
Survival rates				
1-year				
Laparoscopic	88.0%	–	94.2%**	97.6%
Open	78.9%	–	82.4%**	97.3%
3-year				
Laparoscopic	68.8%	88.9%	71.5%**	72.6%
Open	66.9%	86.3%	82.4%**	87.0%
5-Year				
Laparoscopic	–	–	–	64.0%
Open	–	–	–	80.4%
p	0.650	0.660	0.94	0.214

*Postoperative hospital stays

**Disease-free survival rates

## Discussion

This study analyzed the data of patients with GC from a large tertiary hospital and compared LS and open surgery. One hundred and sixty-three patients were included and cholelithiasis was detected in 64 (39.3%) patients. After propensity score matching, LS was found to be superior to open surgery in the operation time, blood loss, drain time, and hospital stay. Meanwhile, there was no significant difference in the cumulative OS and DFS rates between patients who underwent LS and open surgery.

Laparoscopic approaches have been fully developed in the field of surgery due to their advantages of a magnified field of view and convenient operation. Different from the widely recognized application in the field of colonic and gastric cancer, the application of the laparoscopic technique in the field of GC is still controversial. Although LS has not been associated with decreased survival, it is still in the early phase of the adoption curve [14]. Cho et al. [9] retrospectively studied the data of 81 patients with stage T2 GC and selected 19 pairs of patients for comparison by propensity score matched analysis. Compared to the open group, the laparoscopic group had significantly shorter postoperative hospital stay and operation time, and similar OS and DFS rates. Imamura et al. [11] reported 31 patients undergoing laparoscopic gallbladder resection, and concluded that LS was safe and feasible in gallbladder tumors. Kim et al. [15] studied 31 patients with GC and revealed that LS could shorten the postoperative hospital stay without increasing the complications or reducing the survival rates. Lv et al. [16] performed a systematic review and meta-analysis which included 18 studies. They found that patients who underwent LS had less intraoperative hemorrhage and postoperative morbidity, and recovered faster than those who underwent open surgery. The conclusions of the present study are similar to those of the above studies. LS and open surgery demonstrated similar surgical safety and oncological outcomes, but the former was associated with a faster postoperative recovery process.

However, some studies have come to different conclusions. Berger et al. [17] analyzed 27 studies and found a high rate of port-site metastasis in incidental GC. In the clinical practice guidelines for the management of biliary tract cancers launched by the Japanese Society of Hepato-Biliary-Pancreatic Surgery, open surgery was recommended as a rule instead of LS for patients with suspected GC [10]. The reason was that bile spillage, port site recurrence, and incomplete excision of LS could worsen the long-term prognosis. There are two concerns about LS for GC; one is the side effects of pneumoperitoneum on the tumor cells, and the other is the technical difficulty of the procedure. However, most studies against LS for GC were published in the 1990s and early 2000s [18]. With the improvement in surgical skills and technological innovation, relevant studies in recent years have supported the use of LS for the treatment of GC [19–22]. Liu et al. [18] reviewed reports of minimally invasive surgery for GC, including 18 studies on LS and six studies involving robotic surgery. They found that minimally invasive surgery for GC was safe and feasible in selected patients and had similar oncological outcomes as open surgery. Feng et al. [23] studied the prognosis of 1068 patients with GC and found no significant difference in the 1-, 3-, and 5-year survival rates between LS and open surgery. Nakanishi et al. [24] analyzed 14 studies containing 1792 patients and found that LS had better survival rates than open surgery at both T2 and T3 tumor stages. The accumulated experience of surgeons, advancement of laparoscopic instruments, and development of high-definition monitors have all played important roles in improving the safety and feasibility of LS for GC. It is worth mentioning that when comparing LS and open surgery, it is very important to pay attention to the balance of the basic conditions of the two groups of patients. In the present study, the open group had more patients with advanced TNM stage and underwent liver resection than laparoscopic group before propensity score matched analysis. Because patients with earlier stages were more likely to choose LS. Both liver resection and TNM stage were used as predictors for matching, and the two groups were balanced in these aspects after propensity score matched analysis.

LS for GC is in line with the concept of Enhanced Recovery After Surgery (ERAS). ERAS measures can improve the safety and satisfaction of surgical patients and shorten the length of hospital stay [25]. Various conditions in the perioperative period may aggravate the stress and inflammatory response, such as anxiety, surgical trauma, and infection, thus affecting the surgical outcome and long-term prognosis [26]. Trauma is the most important stress factor for surgical patients. It is recommended that the surgery be completed while following the concepts of precision, minimal invasiveness, and damage control to reduce traumatic stress. LS for GC is significantly better than open surgery in terms of damage control and minimal invasiveness. The enlarged field of view and refined operation can effectively reduce surgical trauma and intraoperative blood loss, and accelerate postoperative recovery. The present study also confirmed that patients who underwent LS had a shorter hospital stay and operation time. Because of the above advantages of LS, there have been reports on the application of LS for advanced-stage GC [27].

This study has some limitations. First, the research factors could not be designed in advance due to the characteristics of a retrospective study. Second, the number of patients included was limited because of the low incidence of GC and the single-center setting of the study. Third, the patients were not grouped and analyzed according to the different tumor stages due to the limited patient number. Multicenter, prospective, randomized controlled studies are needed to better clarify the application of LS for GC.

## Conclusion

This study confirmed the safety, feasibility, and oncological outcomes of LS for GC. Compared to open surgery, LS had a shorter operation time, less blood loss, and a shorter drain time and hospital stay. Furthermore, the cumulative OS and DFS rates between patients who underwent LS and open surgery showed no significant differences. LS is worth promoting in patients with GC.

## Data Availability

Research data are available from the corresponding author on reasonable request.
